# Predicting the Role of the Human Gut Microbiome in Constipation Using Machine-Learning Methods: A Meta-Analysis

**DOI:** 10.3390/microorganisms9102149

**Published:** 2021-10-14

**Authors:** Yutao Chen, Tong Wu, Wenwei Lu, Weiwei Yuan, Mingluo Pan, Yuan-Kun Lee, Jianxin Zhao, Hao Zhang, Wei Chen, Jinlin Zhu, Hongchao Wang

**Affiliations:** 1 State Key Laboratory of Food Science and Technology, Jiangnan University, Wuxi 214122, China; 6190111125@stu.jiangnan.edu.cn (Y.C.); wutong9559@outlook.com (T.W.); luwenwei@jiangnan.edu.cn (W.L.); 6180111089@stu.jiangnan.edu.cn (W.Y.); 6190111135@stu.jiangnan.edu.cn (M.P.); jxzhao@jiangnan.edu.cn (J.Z.); zhanghao61@jiangnan.edu.cn (H.Z.); chenwei66@jiangnan.edu.cn (W.C.); 2 School of Food Science and Technology, Jiangnan University, Wuxi 214122, China; 3 Department of Microbiology & Immunology, Yong Loo Lin School of Medicine, National University of Singapore, Singapore 117545, Singapore; micleeyk@nus.edu.sg; 4 International Joint Research Laboratory for Pharmabiotics & Antibiotic Resistance, Jiangnan University, Wuxi 214122, China; 5 National Engineering Research Center for Functional Food, Jiangnan University, Wuxi 214122, China; 6 Wuxi Translational Medicine Research Center and Jiangsu Translational Medicine Research Institute Wuxi Branch, Wuxi 214122, China

**Keywords:** gut microbiome, constipation, machine learning, feature selection, classification model

## Abstract

(1) Background: Constipation is a common condition that affects the health and the quality of life of patients. Recent studies have suggested that the gut microbiome is associated with constipation, but these studies were mainly focused on a single research cohort. Thus, we aimed to construct a classification model based on fecal bacterial and identify the potential gut microbes’ biomarkers. (2) Methods: We collected 3056 fecal amplicon sequence data from five research cohorts. The data were subjected to a series of analyses, including alpha- and beta-diversity analyses, phylogenetic profiling analyses, and systematic machine learning to obtain a comprehensive understanding of the association between constipation and the gut microbiome. (3) Results: The alpha diversity of the bacterial community composition was higher in patients with constipation. Beta diversity analysis evidenced significant partitions between the two groups on the base of gut microbiota composition. Further, machine learning based on feature selection was performed to evaluate the utility of the gut microbiome as the potential biomarker for constipation. The Gradient Boosted Regression Trees after chi2 feature selection was the best model, exhibiting a validation performance of 70.7%. (4) Conclusions: We constructed an accurate constipation discriminant model and identified 15 key genera, including *Serratia*, *Dorea*, and *Aeromonas*, as possible biomarkers for constipation.

## 1. Introduction

Constipation is a common chronic condition worldwide with a complicated etiology; a previous meta-analysis of constipation revealed that the global average prevalence is about 14% [1]. It severely affects the physical and mental health and the quality of life of patients [2]. The clinical diagnosis of constipation is usually made by evaluating the stool form and the associated persistent bowel symptoms, such as the Bristol Stool Form Scale and the Rome IV criteria [3]. The gut microbiome is considered the most important symbiotic microecosystem within a host’s body and there is mounting evidence that the gut microbiome plays a central role in human health and disease [4]. It has also been identified as a crucial determinant of intestinal inflammation and a key player in constipation [5]. The gut microbiome of individuals with constipation tends to differ from that of healthy individuals in terms of taxonomic composition and biological functions. On the other hand, due to the unclear pathogenesis of constipation, it is hard to carry out targeted treatment. The commonly used treatment methods now are usually including changing dietary habits such as increasing dietary fiber intake and using laxatives to promote bowel movements [2]. However, high intake fiber supplements may cause abdominal discomfort such as bloating and fullness, and the impact and safety of long-term use of laxatives are not yet clear, they may cause adverse intestinal symptoms and then cause secondary injuries to patients [6,7]. Because the gut microbiome has a close association to health or constipation, compared with traditional diagnostic standards, the gut microbiome can provide new insights into constipation interventions while realizing discrimination, such as dietary-based microbiome intervention and probiotic therapy [8]. Non-drug treatment options may help to reduce or avoid patient health damage caused by long-term drug use and maintain intestinal homeostasis. Thus, it is valuable to analyze the influence of gut microbiome signatures on constipation and then clarify constipation etiology, and design and evaluate gut microbiota-based interventions to alleviate constipation.

Next-generation sequencing technologies have enabled the study of the gut microbiota in a culture-independent manner, which has yielded glimpses into the complex and incompletely understood interactions between the gut microbiome and its host. To investigate the associations of the gut microbiome with host health and disease, metagenome-wide association studies have begun to explore gut microbiome alterations in constipation, adiposity, diabetes, inflammatory bowel disease, colorectal cancer, and many other conditions [4]. One study found that the α-diversity of the gut microbiome of patients with constipation was higher than that of normal individuals, which suggests that such patients have a complex gut microbiome [9]. Similarly, the β-diversity of the gut microbiome has been observed to differ significantly between patients with constipation and normal individuals [10]. Further, the abundances of different taxa were also associated with constipation. For example, the Firmicutes to Bacteroidetes ratio was considered to characterize intestinal dysbiosis; Bacteroidetes has a higher abundance in the constipated patient and Firmicutes correlated with intestinal transit [10,11]. The relative abundances of Ruminococcaceae and *Akkermansia* were also found to be higher in the patients than in normal individuals [12]. However, most prior studies have focused on a single disease population and a matching control, and very few have integrated data from multiple populations or incorporated data from other studies. These limitations hinder our ability to clarify the robustness of microbiome–disease associations and obscure our understanding of the potential mechanisms by which the microbiome contributes to constipation.

The robustness of microbiome–disease associations can be assessed by a meta-analysis of data integrated from all relevant investigations [13]. A meta-analysis based on large-scale datasets would be an effective approach to identify associations that are consistent across studies and are thus less likely to be due to biological or technical confounders. Next-generation DNA sequencing technologies have been used extensively, which may enable a meta-analysis to reveal association patterns common to independent studies. For example, a meta-analysis of 16S rRNA gene amplicon data has revealed that the originally reported associations between the taxonomic composition of the gut microbiome and obesity were inconsistent across studies and showed only weak statistical significance [4]. A meta-analysis of microbiome data can also improve the prediction capabilities of taxonomic profiles for several diseases. These studies highlight the importance of data integration in contributing to our understanding of the role of the gut microbiome in health and disease.

A deluge of metagenomic data about the human microbiome has been generated, such as those from the Human Microbiome Project and the American Gut Project (AGP), but obtaining biologically and clinically meaningful mechanistic insights from these data remains a major challenge. Machine learning offers next-level analyses that allow the development of new perspectives and novel hypotheses about the human gut microbiome [14]. One study established a machine-learning model using the random forest method to classify constipation status, which yielded an area under the curve (AUC) value of 82% [15] and provided ways to obtain information regarding the physiological and metabolic characterization of the human microbiome. Although the power of machine-learning algorithms is attracting increasing attention, some limitations remain in previous studies. As most of the previous studies have focused on a single cohort study or a relatively small dataset, the predictive models have been typically designed and validated on the same cohort. Moreover, initial attempts were limited to a handful of predefined features, as opposed to extracting high-dimensional and mineable features via high-throughput data-mining algorithms [13]. Hence, the cohort heterogeneity among different studies may limit the reliability and generalizability of these predictive models. Because modern machine-learning technology has yet to be well utilized, few data are available on human microbiome features, limiting their use in identifying constipation status.

In this study, we collected a large panel of constipation data samples from the AGP and the Sequence Read Archive (SRA) database. The data were subjected to a series of analyses, including alpha diversity, beta diversity, and phylogenetic profile analyses, to obtain a deeper and more comprehensive understanding of the association between constipation and the gut microbiome. Machine learning based on feature selection and classification evaluation was performed in training and validation cohorts, and independent cohorts were used to evaluate the potential of the gut microbiome as a noninvasive biomarker for constipation.

## 2. Materials and Methods

### 2.1. Data Inclusion Criteria and Data Collection

Most of our raw data came from the AGP [16] and were downloaded from its website. Samples with fewer than 1250 sequences were excluded. The samples with a BMI between 18.5 and 25, no history of antibiotic medication usage, inflammatory bowel disease, and diabetes within one year were defined as healthy samples, and the index named “bowel_movement_quality” in the metadata table of the AGP was used to determine constipation samples. A total of 777 constipation samples and 2138 healthy samples from the AGP were included in this study. In addition to the AGP data, data from some other studies that met the following inclusion criteria were included to expand the scale of the data: (1) the study focused on constipation and the gut microbiome, and (2) the study evaluated 16S rRNA sequence data, and the raw data could be downloaded from a public database. Based on Pubmed and the search formula (((“constipation”[All Fields]) OR (“astriction”)[All Fields] AND (“microbiome”[MeSH Terms] OR “microbiome”[All Fields])), the sequence data of four extra studies were included in this study with the SRA numbers ERP012611, SRP109879, SRP116968, and SRP169528 [17,18,19,20].

To estimate the reliability and generalizability of the predictive models, we also collected 150 fecal samples from our previous study cohort, including 73 healthy samples and 77 constipation patients with less than three bowel movements per week [21]. All samples included in this study had not been diagnosed with diabetes, inflammatory bowel disease, or irritable bowel syndrome.

### 2.2. Bioinformatic Processing

The SRA format files were converted to fastq format files using the SRA Toolkit. The paired-end read files were combined using FLASH v1.2.7 [22]. To normalize the sequence data collected from different resources while avoiding excessive losses due to differences in data quality, all sequences were filtered to ensure that 80% of the sequences met the quality score (Q) threshold of >20. Because different regions of these sequences were amplified, the operational taxonomic units (OTUs) were generated using the usearch_global method in Vsearch v2.8.0 [23]. The AGP used the Greengenes 13_8 database as the reference genome. However, the database was too old to produce a precise result. Therefore, we used the latest Silva 16s rRNA v138 database as the new reference genome, to produce a more precise result. The alpha-diversity indices (Chao1, observed OTUs, Shannon, and PD whole tree) and beta-diversity matrix were calculated using the QIIME 1.9.1 pipeline [24].

### 2.3. Gut Microbiome Diversity Analysis

To explore the differences in α-diversity between healthy and constipation samples, the Wilcoxon rank-sum test was performed using the “wilcox.test()” function in the basic package of R-3.4.4, with the *p*-value adjusted based on the FDR using the “p.adjust()” function in the basic package of R. For β-diversity, permutational multivariate analysis of variance (Permanova) and a PCA based on weighted Unifrac, unweighted Unifrac, and Bray–Curtis distance matrices were performed using the “adonis()” and “prcomp()” function of the vegan and psych package, respectively [25,26]. The result was plotted using the ggbiplot package in R-3.4.4. The unweighted and weighted Unifrac distance matrices were calculated using the QIIME pipeline, and the Bray–Curtis distance matrix was calculated based on the genus-level OTU table using the “vegdist()” function of the vegan package in R [25].

### 2.4. Taxonomic Analyses

To compare phyla between the two groups, the Firmicutes/Bacteroidetes ratio was calculated and compared using the Wilcoxon rank-sum analysis. To identify the significant differences in genera between the two groups, the balances algorithm provided by the selbal package of R was used to analyze whether balances of particular gut microbial genera could discern constipation phenotype [27].

### 2.5. Microbial Co-Abundance Network Construction

To have a better understanding of the association between the gut microbiome and constipation, the Weighted Gene Correlation Network Analysis (WGCNA) package of R [28] was used to construct a co-abundance network based on the normalized OTU relative abundance data. In total, 192 (6.3%) samples were removed as outliers according to the sample similarity clustering result. After calculating the beta value which satisfies the scale-free topology criteria, a recommended soft threshold was selected (beta = 6) and constructed an unsigned network. The Kendall coefficient was used to calculate the correlation between eigenOTU and binary constipation phenotypes. The significance of the correlation was obtained through the Student asymptotic *p*-value.

Visualization of the co-abundance network modules was performed using the Gephi (0.9.2). Among the modules, a grey module was discarded, OTUs marked with ‘grey’ represented not belonging to any modules and were not associated with the phenotype. In addition, inter-modular connectivity weights greater than the threshold (weight > 0.02) were shown.

### 2.6. Data Normalization

Before constructing the machine-learning model, data normalization was performed to ensure that all features belonged to the standard normally distributed data, because the performance of the model would be affected by variations in the range of features. The following formula was used to normalize the features:

(1) $$v^{\prime }\;=\frac{V-\mu }{s}$$

*V* was the original value, *μ* was the mean value of relative abundance in the corresponding genera, and *s* was the standard deviation of the corresponding genera’s abundance.

### 2.7. Feature Selection

Many features were included in this study, which would generally cause a negative effect in the model. Although several previous studies have attempted to perform such feature selection, they used only a few feature-selection methods. In our study, we used as many feature-selection methods as possible to obtain a more comprehensive understanding of feature selection in metagenomics studies. In total, we used nine feature-selection methods, namely a *t*-test, the Wilcoxon rank-sum test, the Mann-Whitney test, a Chi-squared test (chi2), the F-test, the mutual information test, a logistic regression, a Lasso regression, and a random forest. For the statistical significance tests, the features with *p*-values greater than 0.05 were excluded. For the chi2 analysis and F-test, only the top 10% of features were kept after the analysis. For logistic regression and random forest, the features whose importance was more than the mean of importance were kept, and the threshold for Lasso regression was 1 × 10^−5^.

### 2.8. Model Construction and Grid Search

Before the model construction, it was essential to ensure that the sample size was adequate to produce a reliable result. Therefore, a learning curve was generated to explore the relationship between the sample size and model performance.

To further our understanding, nine machine-learning models, namely k-nearest neighbors (kNN), Support vector machine (SVM), Decision tree (DT), Random forest (RF), Adaptive boosting (ADA), Naive Bayes (NB), Gradient boosted regression trees (GBRT), Logistic regression (Log), and Least absolute shrinkage and selection operator (Lasso), were constructed based on the feature selection mentioned before. For each model, fivefold cross-validation was used for performance evaluation and the AUC value was used as the performance indicator. The mean and standard error of AUC values were reported. To estimate the performance of the feature-selection method, the machine-learning models that lacked a feature-selection method were evaluated as the baseline models.

A grid search of the different parameters of each model was performed to determine the best parameters of the selected model and thus improve its performance. For the Lasso model, the inverse of regularization strength and the maximum number of iterations were adjusted; for the SVM model, the gamma and C were adjusted; for the RF model, the number of weak learners and the split criterion function were adjusted; for the GBRT model, the number of weak learners and the learning rate were adjusted. Each model was also subjected to fivefold verification to avoid random errors.

All machine-learning models were established on the Scikit-Learn pipeline [29] in Python using the neighbors.KneighborsClassifier(), svm.SVC(), tree.DecisionTreeClassifier(), ensemble.RandomForestClassifier(), ensemble.AdaBoostClassifier(), naive_bayes.GaussianNB(), ensemble.GradientBoostingClassifier(), linear_model.LogisticRegression() for the corresponding models.

To identify the potential microbial markers of constipation, we used the recursive feature elimination (RFE) algorithm combined with fivefold cross-validation to determine the relationship between the number of features and the performance of the model. This algorithm was based on the feature-importance scores of the model. It pruned the least-important feature and repeated the process of discarding features until the target number of features was reached. The feature_selection.RFECV() from the Scikit-Learn pipeline was used to implement the algorithm.

## 3. Results

### 3.1. Characteristics of Study Population

**Figure 1:**
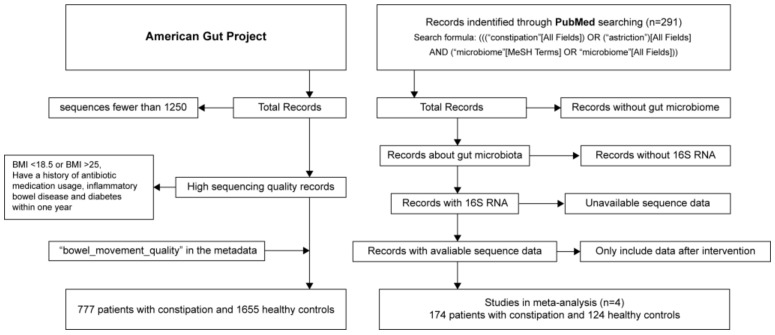
Search and selection of studies.

Data collection from different resources resulted in the inclusion of a total of 918 patients with constipation and 2138 healthy controls from five studies in this meta-analysis. Most of the gut microbiome data were from the AGP, i.e., 777 and 2138 of the total patients with constipation and total healthy controls were from the AGP, respectively. Further, data of 141 patients with constipation were collected from the SRA database, with these deriving from studies with the SRA numbers ERP012611, SRP109879, SRP116968, and SRP169528 (Figure 1). In the model-establishment phase, the gut microbiome data samples from the public database were used to train the machine-learning models. These samples were divided into two parts: 70% of the samples were used as the training datasets to train the models, and 30% of the samples were used to evaluate the performance of the models. In the validation phase, data from 77 patients with constipation and 73 healthy controls were collected by our laboratory to validate the classification efficacy of machine-learning models. As the gut microbiome data included in this study were from different studies, we were able to provide an explanation for the shift in the gut microbiome of patients with constipation from that of the healthy controls.

### 3.2. Gut Microbial Diversity in Patients with Constipation

**Figure 2:**
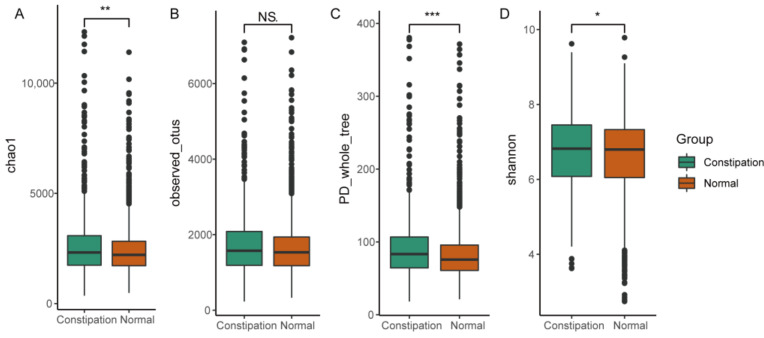
All gut microbiome diversity indices, except the observed operational taxonomic units, increased in patients with constipation (**A**–**D**). Statistical significance between groups is indicated by NS, *, **, ***, corresponding to *p* > 0.05, <0.05, <0.01 and <0.001 respectively.

**Figure 3:**
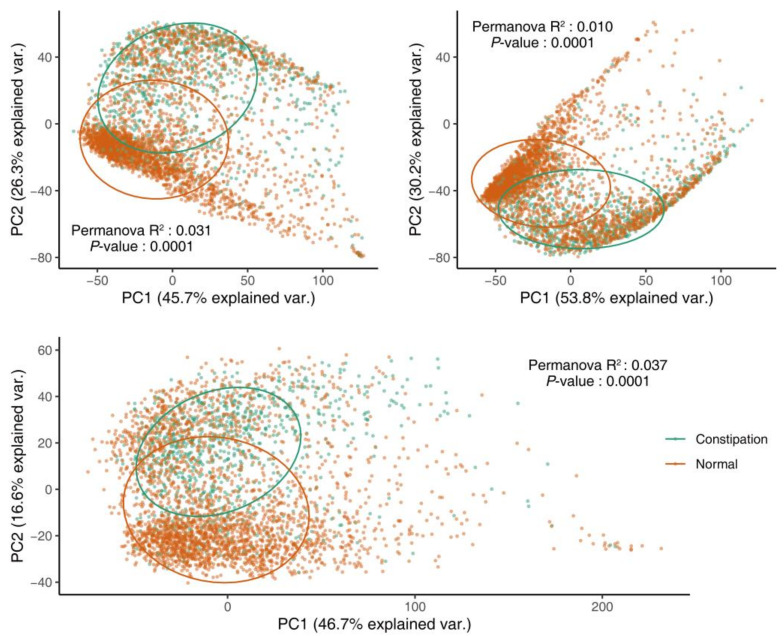
Principal component analysis based on Bray-Curtis, unweighted Unifrac, and weighted Unifrac distance matrices showed significant difference in the β-diversity of the gut microbiome between patients with constipation and healthy controls.

To explore the alteration of gut microbial diversity in patients with constipation, four α-diversity indices, namely the Chao1 index, the phylogenetic diversity (PD) whole tree, observed OTUs, and the Shannon index, were estimated using the Quantitative Insights Into Microbial Ecology (QIIME) pipeline. The Wilcoxon rank-sum analysis was performed to evaluate the differences in α-diversity between patients with constipation and healthy controls. The fecal microbial diversity, as estimated by the Chao1 index, the PD whole tree, and the Shannon index, was significantly higher in the patients than in the controls (Figure 2). To estimate the β-diversity, permutational multivariate analysis of variance (Permanova) and principal component analysis (PCA) based on unweighted Unifrac, weighted Unifrac, and Bray-Curtis distance matrices were performed to explore the microbiome space between the patients and controls. In addition, the results indicated that constipation could cause significant differences in gut microbiome (Figure 3).

### 3.3. Phylogenetic Profiles of the Gut Microbiome of Patients with Constipation

**Figure 4:**
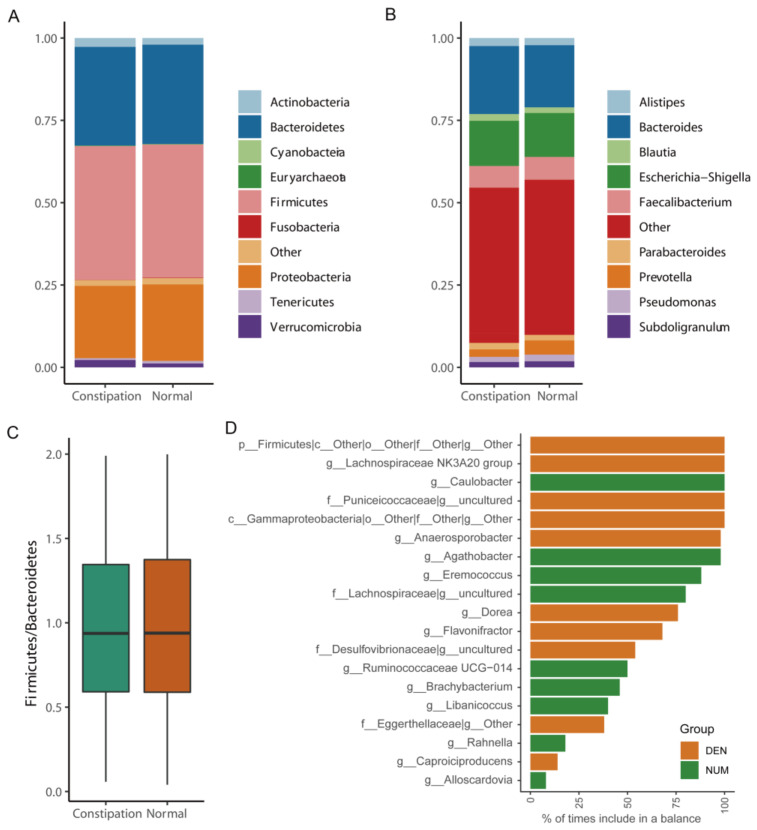
Differences in the gut microbiome composition at the phylum level (**A**) and genus level (**B**) between patients with constipation and healthy controls. The Firmicutes:Bacteroidetes ratio of the gut microbiome did not differ significantly between the two groups (**C**). The balance selected genera that significantly differed between the two groups (**D**).

The bacterial phyla Bacteroidetes, Firmicutes and Proteobacteria, together accounting for up to 90% of sequences on average, were the three dominant populations in the two groups. The average compositions of the bacterial community at the phylum and genus levels are shown in Figure 4A,B, respectively. The ratios of Firmicutes to Bacteroidetes in the two groups were calculated and compared using the Wilcoxon rank-sum analysis. As shown in Figure 4C, the ratios did not differ between two groups. To identify key OTUs, the relative abundances were compared using the balances algorithm. The cross-validation in balances selection demonstrated that a total of 19 genera were identified as key lineages associated with constipation, including Lachnospiraceae, *Agathobacter*, *Dorea,* and Ruminococcaceae (Figure 4D).

### 3.4. Microbial Co-Abundance Network Modules and Constipation Associations

In order to future detect the interaction between different gut microbial OTUs and their potential association with constipation, WGCNA was applied to construct OTU co-abundance network and identify key microbial network modules which were correlated with constipation. Among all genera, 210 OTUs were identified as critical nodes in the co-abundance network.

**Figure 5:**
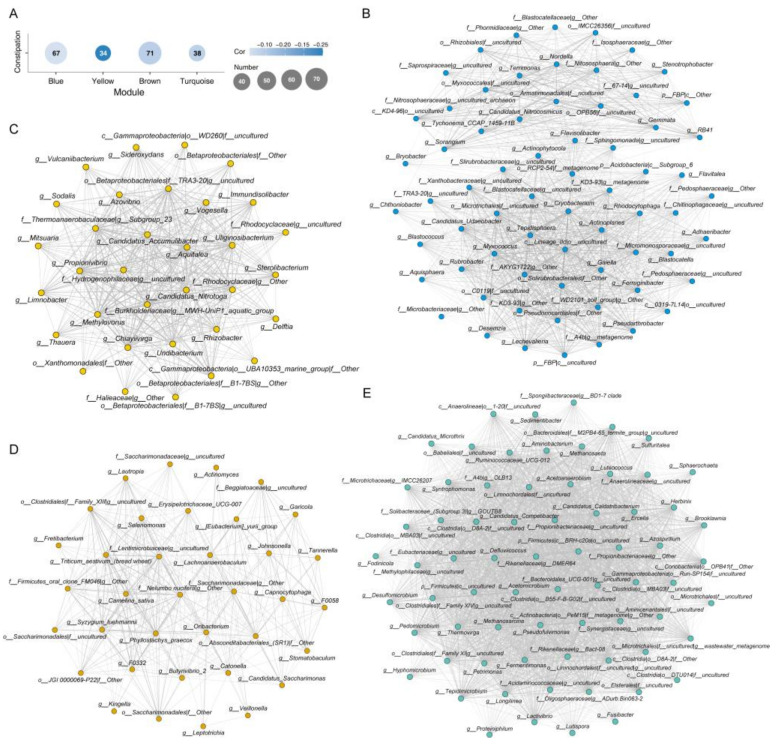
The number of genera and Kendall correlation of clustered modules (**A**). The network modules significantly related to constipation and highly interaction genera were indicated. The cluster of Blue (**B**), Yellow (**C**), Brown (**D**) and Turquoise (**E**).

After module clustering, four network modules that were significantly associated with constipation had been identified (*p* < 0.05) and the number of genera included in each module ranges from 34 to 71 (Figure 5A). Although these modules contained different taxa, phylogenetically relevant OTUs tended to cluster in the same module. Actinobacteria, Bacteroidetes, Proteobacteria and Firmicutes were the dominant phyla in the network (Figure 5B–E).

The results showed that the yellow module demonstrated a significant negative correlation with constipation (Kendell correlation coefficient = −0.25, *p* < 0.001) (Figure 5C); in this module, almost all of its nodes were composed of Proteobacteria, Rhodospirillaceae, and Burkholderiaceae family were the dominant taxa. Particularly, *Rhodocyclaceae Candidatus Accumulibacter* potentially was the regulator within this module (TaxaSignificance = 0.16, Module Membership = 0.93) and played a key role in constipation.

### 3.5. Detection of Constipation Based on the Gut Microbiome

**Figure 6:**
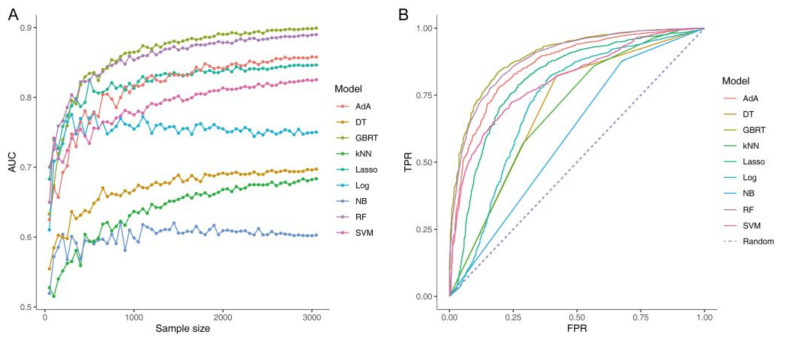
Association between the sample sizes and the area-under-the-curve values of the classifier models (**A**). The receiver operating characteristic curve of all of the models constructed based on the original datasets (**B**).

To explore whether the scale of datasets would affect the performance of classifier models, all of the machine-learning models were trained on 50 subsets by stratified random sampling, with the size of each subset increasing at the same ratio. The performance of each model was evaluated by the receiver operating characteristic curve, which was relatively low and unstable when the sample size was less than 1000 (Figure 6). The performance and sample-size curve tended to plateau with an increase in sample size, which suggested that the data scale used in this study was sufficient to produce a reliable result.

**Figure 7:**
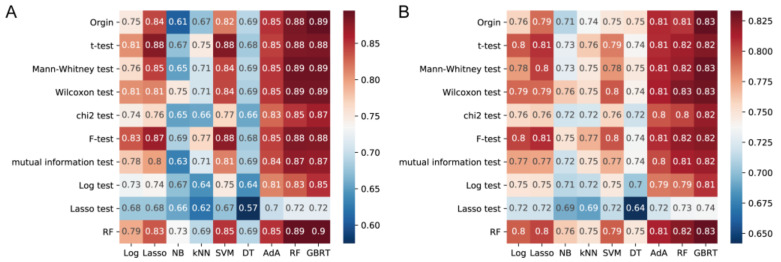
Cross-validated area-under-the-curve values (**A**) and predictive performance of 90 models on test (**B**) datasets. Abbreviations: kNN, k-Nearest Neighbors; SVM, Support vector machine; DT, Decision tree; RF, Random forest; AdA, AdaBoost; NB, Naïve Bayes; GBRT, Gradient tree boosting tree; Log, Logistic.

To illustrate the discriminating value of the fecal microbiome for constipation, a series of machine-learning algorithms, including the AdA, DT, GBRT, kNN, Lasso, Log, NB, RF, and SVM, were constructed from the original OTU table, and their performance was estimated based on their AUC value and predictive accuracy (Figure 7A,B). In the training cohort, the models except Log, kNN, DT, and NB showed a favorable performance with an average AUC value of more than 80%. In particular, the AUC value of the GBRT model was 89.0%, which was the highest among all of the models based on the original datasets (Figure 7A). In addition, it showed the same trend on the test set, in which the GBRT model had the highest predictive performance (Figure 7B).

To improve the predictive performance and provide cost-effective predictions, nine feature selection methods, namely the *t*-test, Wilcoxon test, Mann-Whitney test, chi2 test, F-test, mutual information test, Log test, Lasso test, and RF were performed to select important OTUs from high-dimensional feature space to classify. The subsets of features obtained by different methods exhibit certain similar distribution patterns, which contain overlapping common colony structures (Supplemental Figure S1, Table S1). Subsequently, different feature selection methods and machine learning algorithms combinations were examined for their AUC value and predictive accuracy (Figure 7A,B). Most feature selection methods can be implemented to varying degrees while reducing feature dimensions without affecting performance, especially for tree-based models. Among all of the classifiers, the RF-GBRT model had the highest AUC value (90.0%; Figure 7A).

### 3.6. Validation and Tuning the Parameters of Classifier Models for Constipation

In the validation phase, data from 73 healthy controls and 77 patients with constipation collected by our laboratory were used to estimate the reliability and generalizability of the predictive models and the F-Lasso, T-SVM, RF-RF, RF-GBRT, Chi2-GBRT, and Log-GBRT models were selected. Grid search was performed to determine the best parameters of each model and thus improve their performance.

The verified AUC of most models except RF-RF improved after the grid search (Table 1), which proved that the fine-tuning of a model’s parameters affects its performance. After the optimization of GBRT-based models, their validation performances were all significantly improved (from 49.9%, 62.7%, and 65.1% to 55.5%, 70.7%, and 70.8%, respectively. *p* < 0.05). In sum, after the feature selection and model hyperparameter adjustment, the subset of features obtained using chi2 combined with the GBRT model (chi2-GBRT) showed the best performance in this study, which indicated their greater reliability and generalizability, in addition to their high classification efficacy for constipation.

### 3.7. Identification of Microbial Markers for Constipation

**Figure 8:**
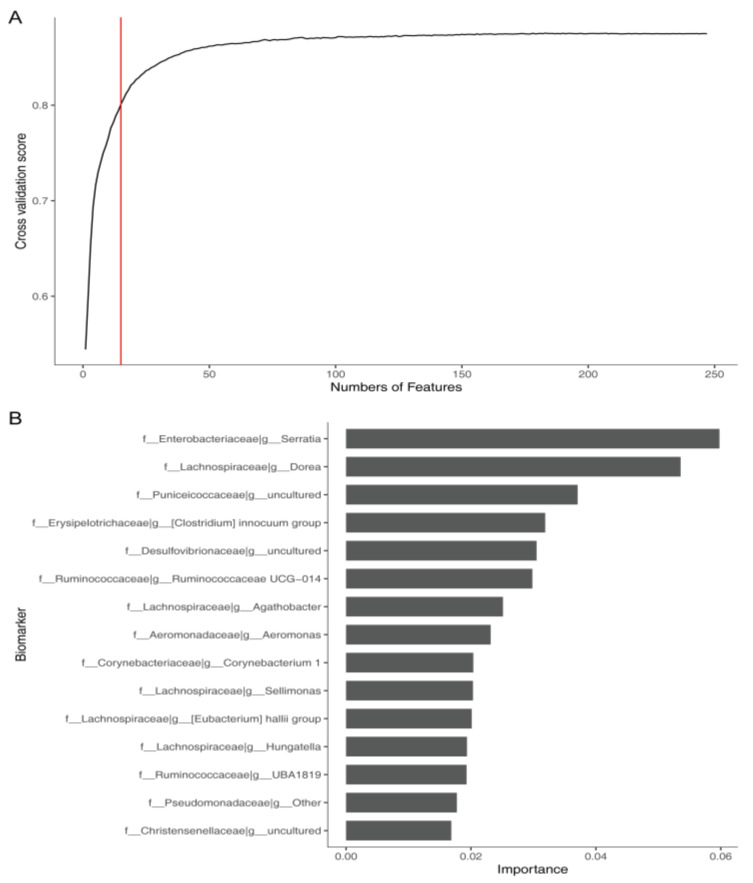
Relationship between the numbers of features and cross-validation scores (**A**). The top 15 genera were selected as the potential biomarkers of constipation (**B**).

To detect unique microbial markers for constipation, we conducted a five-fold cross-validation on chi2-GBRT by plotting the relationship between the number of features and the performance of the model. The results from the RFE algorithm indicated that the performance of the model exceeded 80% when the number of features was 15. Thus, the 15 genera determined based on the feature importance of the chi2-GBRT model, including *Serratia*, *Dorea*, *Agathobacter*, *Aeromonas,* and *Hungatella*, were selected as the optimal marker set (Figure 8A) and are listed in Figure 8B. These genera may provide more information to enable the detection of constipation based on the gut microbiome, and could thus be considered as the potential biomarkers of constipation (Figure 8B).

## 4. Discussion

In this study, by integrating a large panel of gut microbiome features and machine-learning techniques, we established an accurate and reproducible classifier model to detect patients with constipation. The robustness and generalizability of microbiome–disease associations are affected by a wide range of confounders, including population and investigation factors [30]. These study effects may confound the discovery of microbiome signatures that robustly indicate disease status, especially when the data are collected from only a single population or investigation. The integration of metagenomic data in this work enabled comparison of the differences in gut microbiome composition in constipation. Crucially, this efficient classifier was developed using cross-validation and then tested on a new validation cohort, which bolstered its reliability and generalizability.

Like data from any high-throughput data-mining field, metagenomic data are high-dimensional, sparse, skewed with a nonnormal or unknown distributions and have interdependencies. These key characteristics present a challenge for computational and statistical analyses. Thus, there is a need for better algorithmic tools for efficient computation of biological phenotypes. We proposed a series of methods for selecting important OTUs from high-dimensional microbiome data. These feature-selection methods not only improved the predictive performance of the machine-learning models, but also provided faster, cost-effective predictions [31]. In our study, we systematically evaluated multiple feature-selection classification methods, and based on a combination of optimized feature selection method and machine learning algorithm, our machine-learning classifier with the highest predictive performance was formed.

Logistic regression has been widely used for biomarker selection in high-dimensional data, as it requires fewer restrictive assumptions [32]. In this case, the features need not be normally distributed and linearly related to the class or equal in terms of the variance and covariance across groups. Therefore, logistic regression may be preferable for use when the data distribution is nonnormal, or the group sizes are unequal. Chi-square is a simple and general algorithm that can automatically select a proper chi2 value, determine the intervals of a numeric attribute, and select features according to the characteristics of the data [33]. It guarantees that the fidelity of the training data remains after chi2 is applied. The empirical results from both the test and validation datasets showed that logistic regression and chi2 are useful and reliable tools for data discretization and feature selection. Their application affords dimension reduction (feature screening) and prevents over-fitting in the process of training the model. GBRT has become a sought-after model for data scientists in recent years, due to its efficiency and effectiveness in solving practical prediction and classification problems [34]. The combination of these valuable properties meant that logistic or chi2 regression and the GBRT classifier performed excellently in our microbiome analysis.

An important consideration is which taxa contribute to the constipation-prediction model. In this study, we identified a set of top-ranking microbial markers with high feature-importance scores, such as the genera *Serratia*, *Dorea*, *Aeromonas*, and *Hungatella*. A study reported that the fecal abundance of *Serratia marcescens*, a bacterial species that works with other microbial species to form robust biofilms capable of exacerbating intestinal inflammation, was much higher in patients with Crohn’s disease than in healthy controls [35]. The alteration of *Aeromonas* abundance may be important in the pathophysiology of inflammatory bowel disease and its treatment [36]. After fecal microbiota transplantation therapy for chronic intractable constipation, analysis of the recipient’s fecal microbiome revealed a significant abundance of the genera *Hungatella* [37]. A probiotic product could alleviate constipation in mice, mainly by increasing the relative abundance of Ruminococcaceae [38]. Another study found that polysaccharides, oligosaccharides, and a traditional Chinese medicinal formula (Zengye decoction) could alleviate constipation in mice, mainly by decreasing the abundance of *Dorea* at the genus level, which is the predominant gas-producing bacterial genus in the human gut [39]. Moreover, *Dorea* also decreased in pregnant women with digestive diseases and increased in diarrhea-predominant IBS patients [40,41]. All these associations will be subject to further experiment in the subsequent studies. Although the biological behaviors of these microbiome features remain unclear, we posit that these features may still capture the fine characteristics of the gut microbiome and if aimed at establishing and maintaining a healthy balance of microbial community could be a new prevention or therapeutic approach for constipation.

In conclusion, our results demonstrate that the OTU-based taxonomy of gut microbiome combined with machine-learning techniques can identify accurate and generalizable indicators of constipation and that prediction of constipation is most accurate using the chi2-GBRT model. Building on these results, our future work will involve the development of noninvasive microbiome-based tests to determine signs of constipation, and the design and evaluation of microbial interventions to alleviate constipation. In addition, new methods of analysis using amplicons will also be considered in subsequent studies, such as Amplicon Sequence Variants (ASV), which may help improve the accuracy of sequence identity and contain more information.

## Figures and Tables

**Table 1:** The performance of models before and after adjusting the parameters.

	before			after		
	Train AUC	Test AUC	Validation AUC	Train AUC	Test AUC	Validation AUC
F-Lasso	86.8%	84.5%	49.9%	86.9%	84.8%	50.6%
T-SVM	88.1%	83.5%	52.1%	88.4%	84.5%	54.3%
RF-RF	89.4%	89.7%	52.6%	90.3%	90.6%	49.4%
RF-GBRT	89.5%	89.9%	49.9%	90.8%	91.1%	55.5%
Chi2-GBRT	86.5%	86.8%	62.7%	87.3%	87.5%	70.7%
Log-GBRT	85.2%	85.4%	65.1%	85.9%	86.2%	70.8%

## Data Availability

The data presented in this study are openly available in GitHub at https://github.com/hcwang-jn/gut-constipation (accessed on 18 September on 2021).

## Supplementary Materials

The following are available online at https://www.mdpi.com/article/10.3390/microorganisms9102149/s1, Table S1: Biomarkers of gut microbiota associated with constipation obtained by different feature selection methods. Figure S1: Variation in biomarkers obtained by different feature selection methods.
