# Supplementary figures and images for: Predicting the Role of the Human Gut Microbiome in Constipation Using Machine-Learning Methods: A Meta-Analysis
Source: Microorganisms. 2021 Oct 14;9(10):2149. doi: 10.3390/microorganisms9102149 (PMC8539211; doi:10.3390/microorganisms9102149)

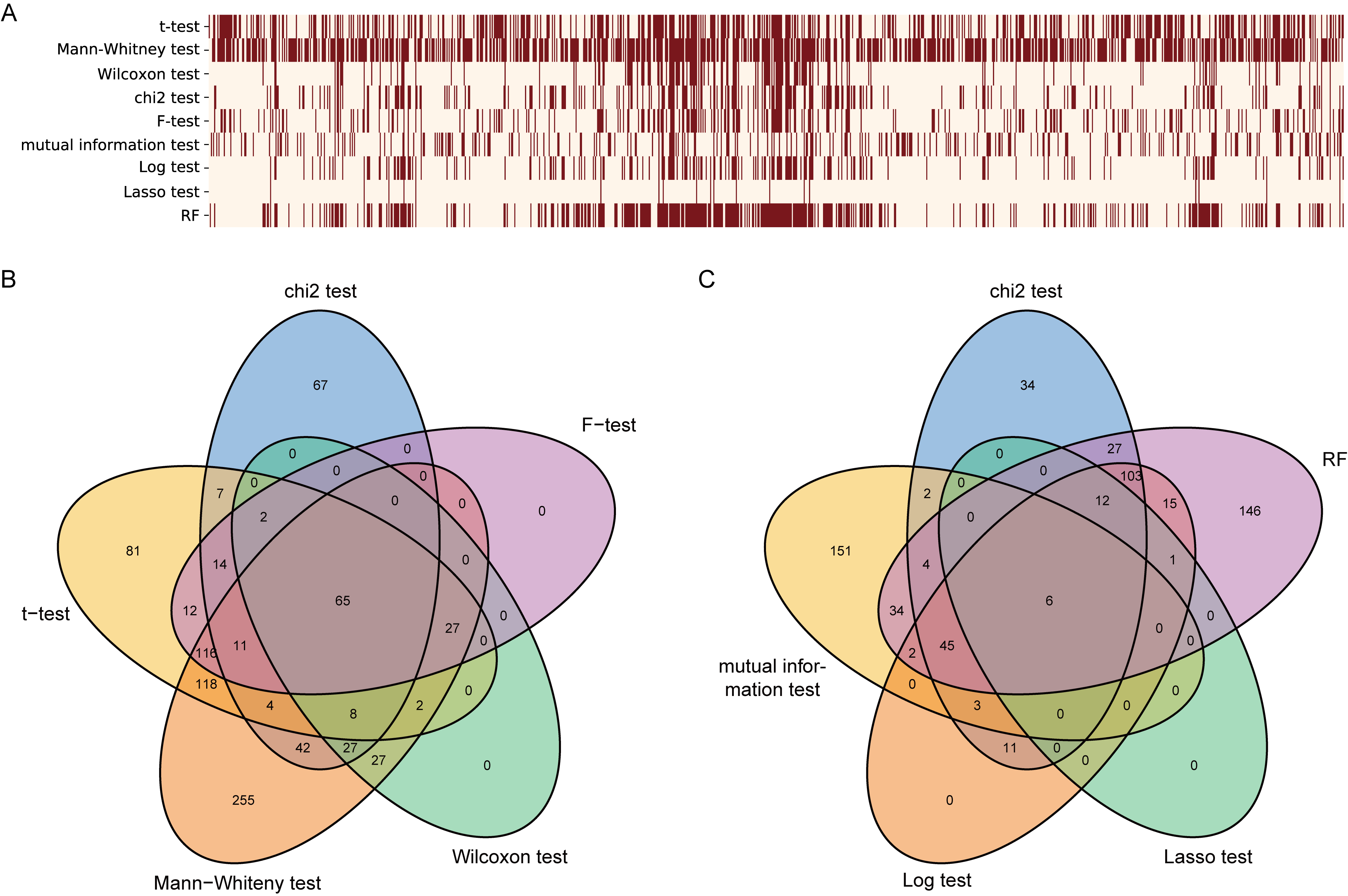

Supplement: Supplementary file 1 [file microorganisms-09-02149-s001.zip › Supplemental_figure_S1.tif]
